# Electrical polarization switching of perovskite polariton laser

**DOI:** 10.1515/nanoph-2023-0829

**Published:** 2024-02-19

**Authors:** Karolina Łempicka-Mirek, Mateusz Król, Luisa De Marco, Annalisa Coriolano, Laura Polimeno, Ilenia Viola, Mateusz Kędziora, Marcin Muszyński, Przemysław Morawiak, Rafał Mazur, Przemysław Kula, Wiktor Piecek, Piotr Fita, Daniele Sanvitto, Jacek Szczytko, Barbara Piętka

**Affiliations:** Faculty of Physics, 49605 Institute of Experimental Physics, University of Warsaw, ul. Pasteura 5, PL-02-093 Warsaw, Poland; 518742 CNR NANOTEC, Institute of Nanotechnology, Via Monteroni, 73100 Lecce, Italy; 518742 CNR-NANOTEC, Institute of Nanotechnology, UOS Rome, SLIM Lab c/o Dip. Fisica, Universit “La Sapienza”, Piazzale A. Moro 2, 00185, Rome, Italy; Institute of Applied Physics, Military University of Technology, PL-00-908 Warsaw, Poland; Institute of Chemistry, Military University of Technology, PL-00-908 Warsaw, Poland

**Keywords:** perovskite, exciton-polariton, lasing, microcavity, spin-orbit coupling, liquid crystal

## Abstract

Optoelectronic and spinoptronic technologies benefit from flexible and tunable coherent light sources combining the best properties of nano- and material-engineering to achieve favorable properties such as chiral lasing and low threshold nonlinearities. In this work we demonstrate an electrically wavelength- and polarization-tunable room temperature polariton laser due to emerging photonic spin–orbit coupling. For this purpose, we design an optical cavity filled with both birefringent nematic liquid crystal and an inorganic perovskite. Our versatile growth method of single CsPbBr_3_ inorganic perovskite crystals in polymer templates allows us to reach strong light–matter coupling and pump-induced condensation of exciton–polaritons resulting in coherent emission of light. The sensitivity of the liquid crystal to external voltage permits electrical tuning of the condensate energy across 7 nm; its threshold power, allowing us to electrically switch it on and off; and its state of polarization sweeping from linear to locally tilted circularly polarized emission.

## 1 Introduction

Development of miniature lasing devices that allow for efficient control over the state of emitted light is one of the main objectives of nanophotonics. In particular, polarization engineering can be achieved using the effective spin–orbit interaction of light that couples the polarization (spin) of a photon with its direction of propagation [1]. Of special interest, for rapidly developing fields focused on light–matter interactions and quantum technologies, are chiral light sources [2], [3], [4], [5] due to their potential in optical trapping, sensing, and communication technologies. The chirality of the emitted light can be imposed by the asymmetric design of the entire structure [6], [7], [8], [9]. A planar cavity can be constructed from chiral mirrors for example based on metamaterials [10], or cholesteric liquid crystals (LCs) [11]. Lasing in structures using chiral organic molecules can exhibit a non-zero degree of circular polarization in the emitted light [12], [13]. In so-called spin lasers circularly polarized emission is obtained from fully homogeneous structures by injection of spin-polarized carriers [14].

However, the tunability of the emission (in terms of light polarization, wavelength, threshold) is difficult to achieve in a single device. Realistic application of optical devices in e.g. classical- and quantum-information processing strongly benefits from the introduction of internal interactions that would allow for tunability of the device through external fields (electric or magnetic). One of the means to introduce strong interactions into an optical structure is to strongly couple a photonic mode with an excitonic dipole. Such coupling can be enhanced by the use of an optical resonator in a form of a microcavity, in which the electric field of a photonic mode is strongly localized and tuned in resonance with the exciton energy [15]. In this strong coupling regime excitons and cavity photons form highly interactive light–matter quasiparticles known as exciton–polaritons. Being composite bosons, with two-component spin structure explicitly related to the circular polarization of the cavity photon |*ψ*_±_⟩ ↔ |*σ* ^±^⟩, and very light effective mass, at high concentrations can undergo a phase transition to a macroscopically populated quantum state referred to as a nonequilibrium Bose–Einstein condensate, characterized by the emission of coherent and polarized light [16], [17], [18], [19], [20]. The macroscopic spin state of the polariton condensate can be described using a pseudospin formalism [15] which explicitly relates to the Stokes vector of the emitted cavity light from the condensate. In addition, exciton polaritons in planar semiconductor microcavities can exhibit superfluidity [21]; long propagation distances and optical switching [22], also optical [23] and electrical switching [24] of chiral emission, and a wide range of spin–orbit coupling phenomena [25], [26], [27], [28].

Exciton polariton condensation, initially investigated predominantly at low temperatures, is today accessible at room temperature thanks to progress in material science [29]. A specific class of materials that have recently gained significant attention for broad optoelectronic applications, and polariton condensation, are perovskites [30], [31], [32], in which the optical response exhibit pronounced excitonic effects [33]. Perovskites have been considered as a promising platform for room temperature polaritonics [34], due to unique phenomena observed in those materials e.g.: strong interactions [35], polariton condensation [36], [37], [38], [39], parametric amplification [40], long distance propagation [41], [42], optical switching [43], and can introduce linear polarization splitting to the cavities [38], [39], [44], [45], [46].

In this work, we introduce inorganic CsPbBr_3_ perovskite monocrystals into an optical microcavity filled with a birefringent LC. The presence of the perovskite allows us to reach the strong coupling regime where polaritons become the new eigenmodes of the system. At high enough incoherent pumping powers, polaritons become stimulated and spontaneously form a macroscopically coherent condensate. Thanks to the sensitivity of the LC to an external electric field, we are able to control the cavity’s optical anisotropy, and subsequently the polarization state of the condensate, using a variable voltage applied across the cavity. In fact, the polariton modes inherit effective photonic spin–orbit coupling (SOC) mechanism coming from the LC material, giving access to distinct 2 × 2 polaritonic spin Hamiltonians including equal Rashba–Dresselhaus SOC [47], [48]. This flexibility not only allows us to all-electrically switch the condensate between different polariton energy branches, but also to gradually divide the condensate into its spin-up and spin-down components, nested in distinct dispersion valleys, resulting in two opposite circularly polarized emission beams exiting the cavity at different angles [49], [50], [51].

**Figure 1:**
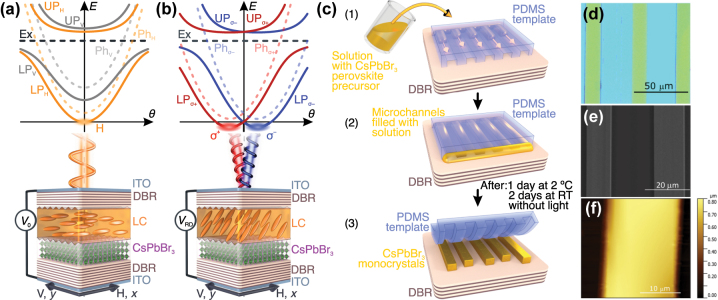
Liquid crystal microcavity polariton laser and synthesis of perovskite monocrystals. Scheme of the device and corresponding dispersion relation of polaritonic modes in two regimes. (a) Without external voltage *V*_0_, anisotropy of the liquid crystal (LC) distinguishes H and V polarized modes, with the condensation occurring at the bottom of H mode. (b) With external voltage *V*_RD_ corresponding to Rashba–Dresselhaus spin–orbit coupling regime the dispersion relation consists of a set of two circularly polarized modes, separated in the emission angle. Condensation occurs simultaneously at the two degenerate minima of the dispersion relation. (c) Schematic of the synthesis of perovskite by solution crystallization. (d–f) Different images of the 500 nm thick CsPbBr_3_ perovskite monocrystals on DBR: (d) optical microscope image, (e) scanning electron microscope image and (f) atomic force microscopy scan.

A scheme of the device is presented in Figure 1. The perovskite crystal is enclosed between two dielectric distributed Bragg reflectors (DBRs). Without the external electric field (Figure 1(a)) optically anisotropic LC molecules distinguish photonic cavity modes with horizontal (H) and vertical (V) polarization. The bare cavity modes exhibit approximately a parabolic dispersion relation versus the emission angle *θ*, proportional to the in-plane wave vector of the cavity modes $k_{\|}=\left(E_{{\text{Ph}}_{\text{H/V}}}/\hbar c\right)\sin\theta$ , plotted in Figure 1(a) with dashed curves. Here, we define $k_{\|}\equiv {\left(k_{x},k_{y}\right)}^{\text{T}}$ with the *z*-direction being along the cavity growth axis. In the strong coupling regime, the cavity modes mix with the excitonic resonance in the perovskite, forming upper (UP_H/V_) and lower (LP_H/V_) polariton modes (plotted with solid curves). Under nonresonant excitation, the polaritons can form a condensate that occupies the bottom of the dispersion relation with lowest losses, in our case, the H-polarized mode.

Depending on the electric bias applied to the ITO electrodes, we can tilt the anisotropy axis of the LC layer. This changes the effective refractive index for horizontally polarized light, which affects the energy of H-polarized modes, while the V-polarized modes remain unaffected. In particular, such tunability can lead to a situation where two distinct longitudinal cavity modes with perpendicular polarizations are almost degenerate. In a specific situation where the almost-degenerate modes are of opposite parity, effective photonic Rashba–Dresselhaus SOC between the modes emerges [47], with two momentum-separated dispersion valleys forming between the circularly polarized modes (Figure 1(b)). When strongly coupled with the exciton the resulting polariton modes preserve polarization splitting of the bare cavity modes and simultaneously gain nonlinear properties and tunability from the exciton admixture.

In this Rashba–Dresselhaus regime, when pumped with laser power above the condensation threshold, the emission from the cavity consists of two circularly polarized beams, which are emitted at a non-zero angle [49], [50], [52]. Here, we additionally utilize the change of the condensation threshold between different regimes to switch on or off the condensate with the external field.

## 2 Preparation of a liquid crystal microcavity with perovskite single crystals

Perovskite single crystals were synthesized directly on DBR substrates by means of a microfluidics-assisted technique on a confined microstructured polymeric template [53], [54] properly modified and optimized to obtain CsPbBr_3_ microcrystals with tailored dimensions. This technique allows for regulating the solvent evaporation rates and hence the supersaturation levels of the perovskite solution confined within a microchannel template. In this way, it is possible to precisely finalize the nucleation process, control the crystal growth, and hence the dimension and quality of the obtained micro-crystals. A sketch of the synthetic process is shown in Figure 1(c): a microchannel polymeric template of polydimethylsiloxane (PDMS) is placed in close contact with the DBR substrate and soaked with 3 μl of a 0.42 M solution of CsPbBr_3_ in dimethyl sulfoxide (DMSO). Due to capillary action, the solution fills the microchannels. Initially, the crystallization process is observed under an optical microscope for the first 10 min after the infiltration of the solution. The surface of the dielectric mirrors is rough because of the polycrystallinity of the sputtered layers. Such a roughness, combined with the confinement of precursor solution inside the microchannels, favors the formation of nucleation centers of the perovskite, and from there the growth of crystals on the mirror surface begins. After the appearance of the first perovskite crystals, the sample is sealed in a small box and left undisturbed at 2 °C for 24 h and then at room temperature for 48 h in the dark under atmospheric conditions. At the end of this process, the PDMS template is removed from the substrate leaving perovskite single crystals on DBR, with the sizes given by the patterned template. The obtained single crystals are of good quality, smooth, and homogeneous. One of the advantages of this technique is that it allows careful modulate the lateral dimensions (width and length) and thickness of the crystals by simply changing the PDMS template. For example, we have developed crystals with lateral dimensions of 15 μm, a length up to 200 μm and a thickness of 500 nm (Figure 1(d–f)) and crystals with lateral dimensions of 100–200 μm with a thickness of 2 μm (see the Supporting Information).

Single perovskite crystals in the bottom dielectric mirror are protected with a 60 nm protective PMMA layer (3 % PMMA solution in anisole) applied using the spin-coating process. A polymer layer SE-130 is applied to the upper mirror (6 pairs of SiO_2_/TiO_2_ layers with ITO). The dielectric mirrors are assembled to form a microcavity with a thickness of several μm, and a birefringent liquid crystal (Δ*n* = 0.3383 at *λ* = 636 nm [55]) is forced into the cavity. ITO electrodes on the microcavity have been soldered to copper wires and connected to driving unit in order to modulate the refractive index of the LC.

## 3 Strong coupling regime

**Figure 2:**
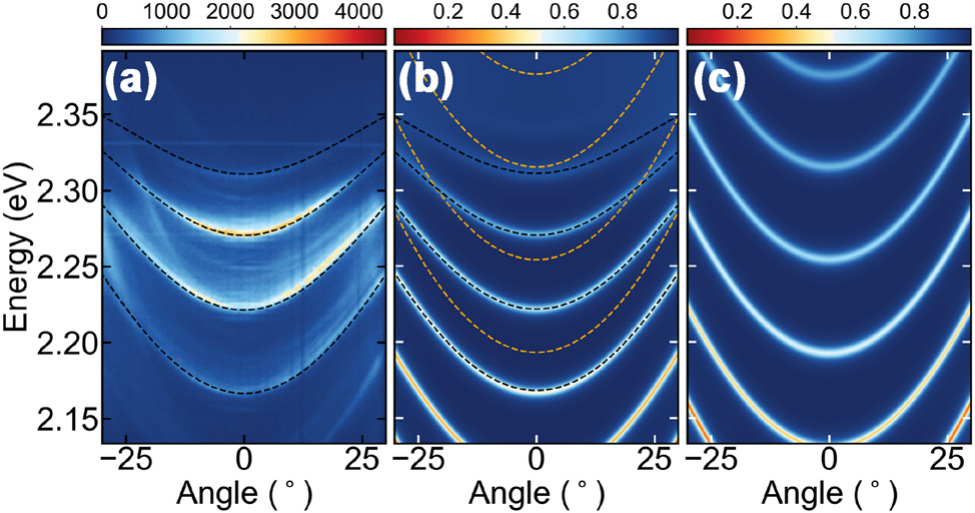
Strong coupling regime in microcavity with CsPbBr_3_ monocrystals. (a) Angle-resolved photoluminescence spectra from the microcavity. The black dashed lines mark the simulated dispersion of the structure presented in (b). (b) Simulated reflectance from the cavity with the perovskite. Orange dotted lines mark dispersion of the bare cavity modes and black dashed lines represent results from the coupled oscillator model. (c) Simulated reflectance from the corresponding structure with perovskite without excitonic resonance (i.e., bare photons).

To demonstrate strong coupling between the perovskite exciton and cavity photon modes, we first used the 2 μm thick perovskite monocrystals. Figure 2(a) presents angle resolved photoluminescence (PL) spectra collected at excitation powers (with a nonresonant pulsed laser) significantly below the condensation threshold. Because of the large thickness of the chosen perovskite monocrystal, the emission spectra consists of multiple polariton modes. Here we show only the lower polariton modes lying below the exciton resonance at *E*_*X*_ ≈ 2.4 eV. The strong coupling regime can be evidenced from the different concavity of the observed polariton dispersion branches as compared to the bare photonic branches (compare black and orange dashed curves in Figure 2(b)). That is, polaritons become heavier as they become more exciton-like. Indeed, the lowest energy polariton branch (2.167 eV at normal incidence) exhibits an approximately parabolic dispersion relation with higher concavity (lighter effective mass) with respect to the higher energy branches which are closer to the exciton line. We note, as a result of high absorption of the perovskite at energies close and above the exciton line, the upper polariton branches cannot be observed accurately enough in the emission.

The experimentally observed dispersion of the modes is in perfect agreement with the numerical simulations. The optical response of a layered, optically anisotropic structure can be calculated using the Berreman method. Figure 2(b) presents simulated reflectance from 4.95 μm thick perovskite enclosed between two SiO_2_/TiO_2_ DBRs using this approach. To simulate the perovskite layer, we used the dielectric function extracted from the ellipsometric data from Ref. [56]. The reflectance minima in the simulated spectra correspond to the polaritonic modes in the structure. Their position was fitted and plotted on top of the photoluminescence results (Figure 2(a)) by black dashed line. The simulated dispersion precisely follows the experimental data.

To show that the observed dispersion of the modes can be considered as a result of the strong coupling between the exciton in the perovskite crystal and the cavity mode, additional numerical modeling was performed. The parameters of the dielectric function of the perovskite allow the removal of the peak corresponding to the excitonic resonance. Simulations performed for such structure lacking the excitonic resonance are presented in Figure 2(c). As expected, the spectrum consists of a series of parabolic bare cavity modes. Their dispersion relation can be used in a coupled oscillator model to mix with the exciton state to obtain the exciton–polariton modes. The lower polariton branches resulting from four independent 2 × 2 oscillator models [57] with exciton resonance at 2.4 eV and consecutive bare cavity modes are presented in Figure 2(b) in black dashed lines. All calculated for the same coupling strength of *ℏ*Ω_R_ = 76.4 meV accurately follow the simulated reflectance minima (Figure 2(b)), as well as the experimental photoluminescence results (Figure 2(a)).

## 4 Polariton condensation in the LC microcavity

**Figure 3:**
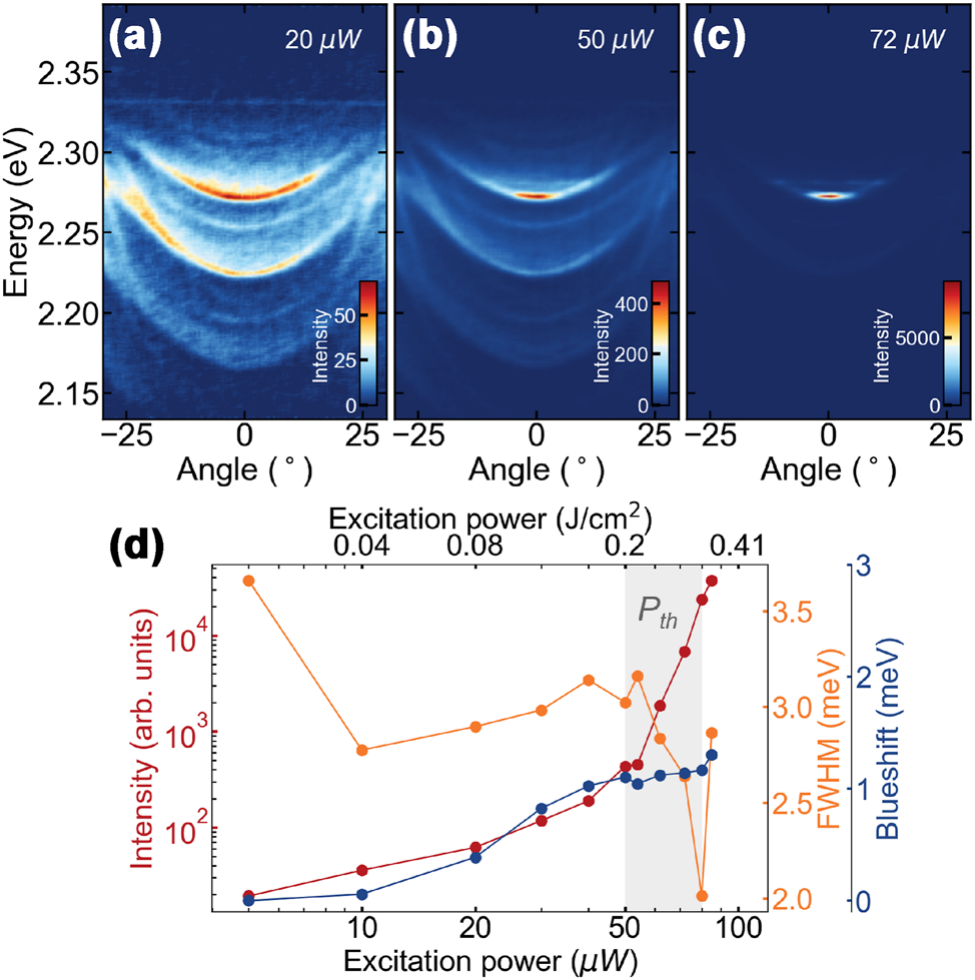
Polariton condensation from CsPbBr_3_ perovskite LC microcavity. (a–c) Angle-resolved photoluminescence spectra with polariton dispersion relation (a) below, (b) at, and (c) above threshold. (d) Polariton emission intensity, FWHM (full width at half maximum) and energy blueshift for increasing excitation power.

The presence of a strong coupling between the perovskite excitons and the cavity modes begs for investigation of nonlinear phenomena in the system. The following experiments are still performed on the 2 μm thick perovskite crystals where we see the clearest formation of polaritons. Figure 3 shows polariton condensation taking place in our perovskite liquid crystal cavity with increasing power, without external electric field. At low powers of non-resonant pulsed excitation (see Methods) the emission is broadly distributed along the polaritonic branches, as presented on angle-resolved PL spectra in Figure 3(a). Consecutive polaritonic modes are visible in the spectrum. With increasing pump power (Figure 3(b and c)) the emission collapses to the bottom of the branch, *k*_‖_ ≈ 0 with the lowest polariton losses (i.e., highest emission intensity below threshold). The observed dependence of the emission intensity, spectral line width, and emission energy with pumping power – presented on a double logarithmic plot in Figure 3(d) – follows the typical behavior for the formation of a polariton condensate. Above the condensation threshold of 72 μW the emission intensity nonlinearly increases, followed by an abrupt decrease of the emission linewidth. The steady increase of the emission energy with the excitation power can be interpreted as a result of polariton-polariton interactions stemming from the excitonic component of the polariton. An interesting observation is the slow change in the condensate energy even around threshold which suggests that most of the blueshift, for the thick crystals, is coming from a saturated background reservoir of excitons.

## 5 Electrical switching of a polariton condensate

**Figure 4:**
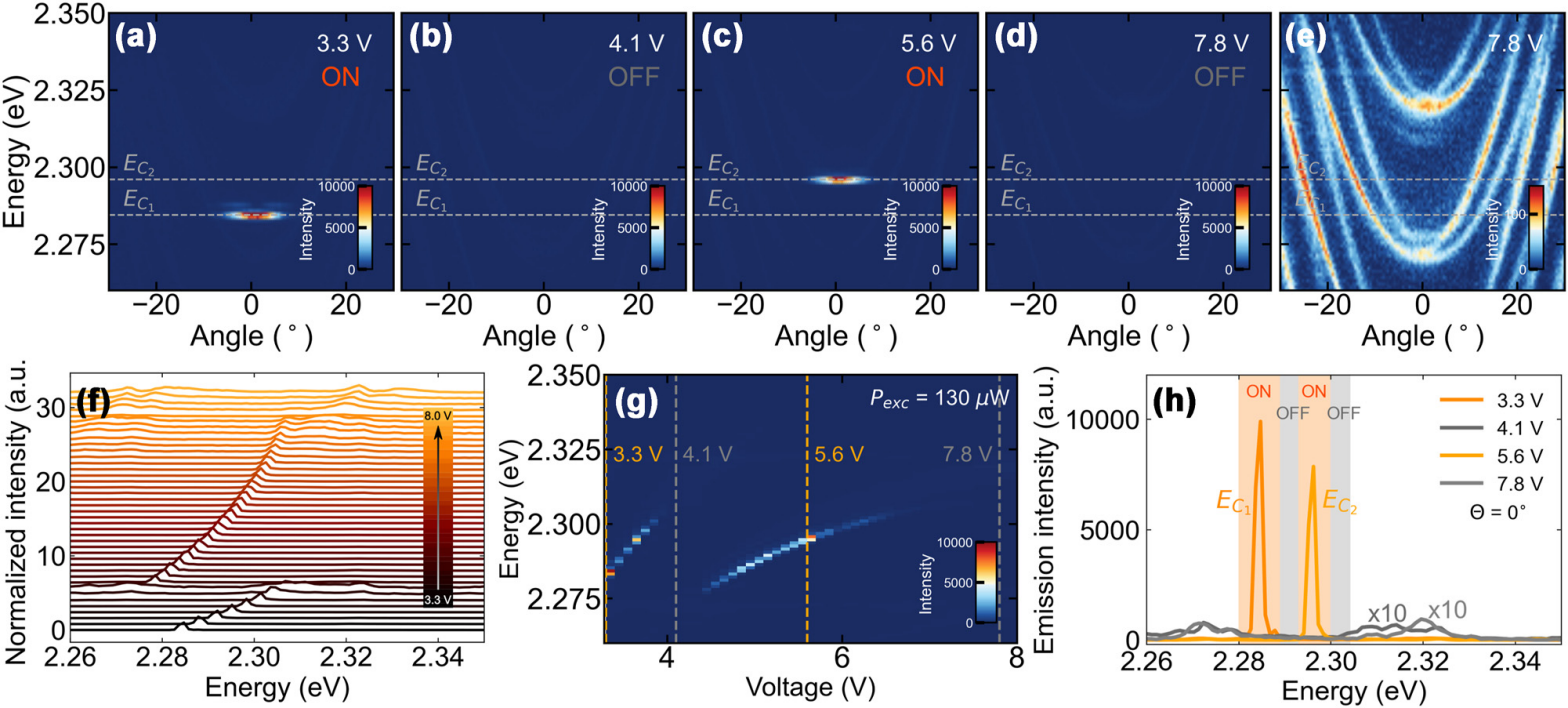
Electrical switching of a polariton condensation emitting in horizontal (H) polarizaton. (a–d) Angle-resolved photoluminescence spectra for different applied voltages with fixed intensity scale. Polariton condensation for 3.3 V (a) and 5.6 V (c). Weak emission spectra for 4.1 V (b) and 7.8 V (d). (e) The same as (d) but with rescaled intensity revealing Rashba–Dresselhaus SOC regime. (f and g) Emission intensity at a normal angle for increasing voltage illustrating smooth energy tunning of polariton condensate. (h) Normal angle emission spectra for (a–d), showing the switching of polariton condensation ON/OFF.

As discussed previously, our optically anisotropic cavity discriminates between orthogonal linearly polarized photonic modes, corresponding to the directions parallel (H-polarized) and perpendicular (V-polarized) to the LC molecular director [47]. In the weak coupling regime, we previously observed that photon lasing occurs preferentially in a H-polarized mode at *k*_‖_ ≈ 0 corresponding to the lowest cavity losses [49]. In the strong coupling regime, we observe the same behavior in the polariton condensate, the energy of which can be tuned by an external voltage applied to the ITO electrodes in the sample. We now work with a sample with 500 nm thick perovskite crystals which depicts greater sensitivity to the electric field while retaining strong coupling. We determined the condensation threshold at 130 μW without the external electric bias. Figure 4(a) presents an angle-resolved PL spectrum just above the condensation threshold power, where most of the emission is concentrated close to *θ* = 0 angle.

Keeping the excitation power constant, we increase the voltage up to 4.0 V and subsequently blueshift the H-polarized polaritonic modes, increasing the condensate energy from 2.276 eV to 2.305 eV (energy tuning of 29 meV in total, or 7 nm), as shown in Figure 4(f and g) presenting the emission spectra at normal incidence. With a further increase of the voltage, the H-polarized polariton mode shifts into the neighbouring V-polarization mode. The emerging photonic Rashba–Dresselhauss SOC between the two modes results in the increase of the condensation threshold power (what is discussed in details in the next section). As the threshold power increases above the excitation power the emission turns off, as shown in Figure 4(b). Above 4.2 V another H-polarized mode approaches the optimum gain spectral range, and the condensate switches back on (Figure 4(c)), and can be further energy-tuned up to 7 V. Once again, above that value when the H-polarized mode approaches the V-polarized one, the emission intensity shuts off (Figure 4(d)). Photonic Rashba–Dresselhaus coupled modes are clearly visible in Figure 4(e) (rescaled Figure 4(d)). Here, for coupled modes, the condensation threshold is higher than the excitation power, and the emission comes from all states along the dispersion relation. The general behavior of the condensate is summarized in Figure 4(f–h), presenting emission spectra at a normal angle for different voltages. Depending on just the amplitude of the external electric field, we are able to energy-tune the emission from the condensate or to switch it off.

## 6 Circularly polarized polariton condensation

Due to the photonic Rashba–Dresselhauss SOC, the condensate emission intensity and energy can not only be tuned with voltage but also its momentum and polarization structure.

The dispersion relation of the bare cavity modes in this regime (dashed lines in Figure 1(b)) can be described by an effective photonic Hamiltonian written in the circular polarization basis [47]:

(1) $${\hat{H}}_{\text{RD}}=\frac{\hbar k_{\|}^{2}}{2m}-2\alpha k_{y}{\hat{\sigma }}_{z},$$

where *m* is the effective mass of the cavity modes, *α* gives the strength of the Rashba–Dreselhauss spin–orbit coupling and ${\hat{\sigma }}_{z}$ is the third Pauli matrix. The dispersion minima split into a pair of valleys at opposite nonzero emission angle **k**_‖_ ≠ 0.

In the strong coupling regime the emerging polariton modes can be described by a Hamiltonian derived from a coherent coupled-oscillator model [50], [58]:

(2) $${\hat{H}}_{\text{SC}}=\left(\begin{array}{cccc} E_{\text{X}} & 0 & \frac{\hbar }{2}\Omega_{\text{R}} & 0\\ 0 & E_{\text{X}} & 0 & \frac{\hbar }{2}\Omega_{\text{R}}\\ \frac{\hbar }{2}\Omega_{\text{R}} & 0 & \frac{\hbar k_{\|}^{2}}{2m}-2\alpha k_{y} & 0\\ 0 & \frac{\hbar }{2}\Omega_{\text{R}} & 0 & \frac{\hbar k_{\|}^{2}}{2m}+2\alpha k_{y} \end{array}\right),$$

written in the canonical spin-up and spin-down exciton basis which couples to right-hand and left-hand circularly polarized photons, respectively. Here, *ℏ*Ω_R_ gives the exciton–photon coupling strength (i.e., Rabi frequency) and *E*_X_ is the spin-degenerate exciton energy.

**Figure 5:**
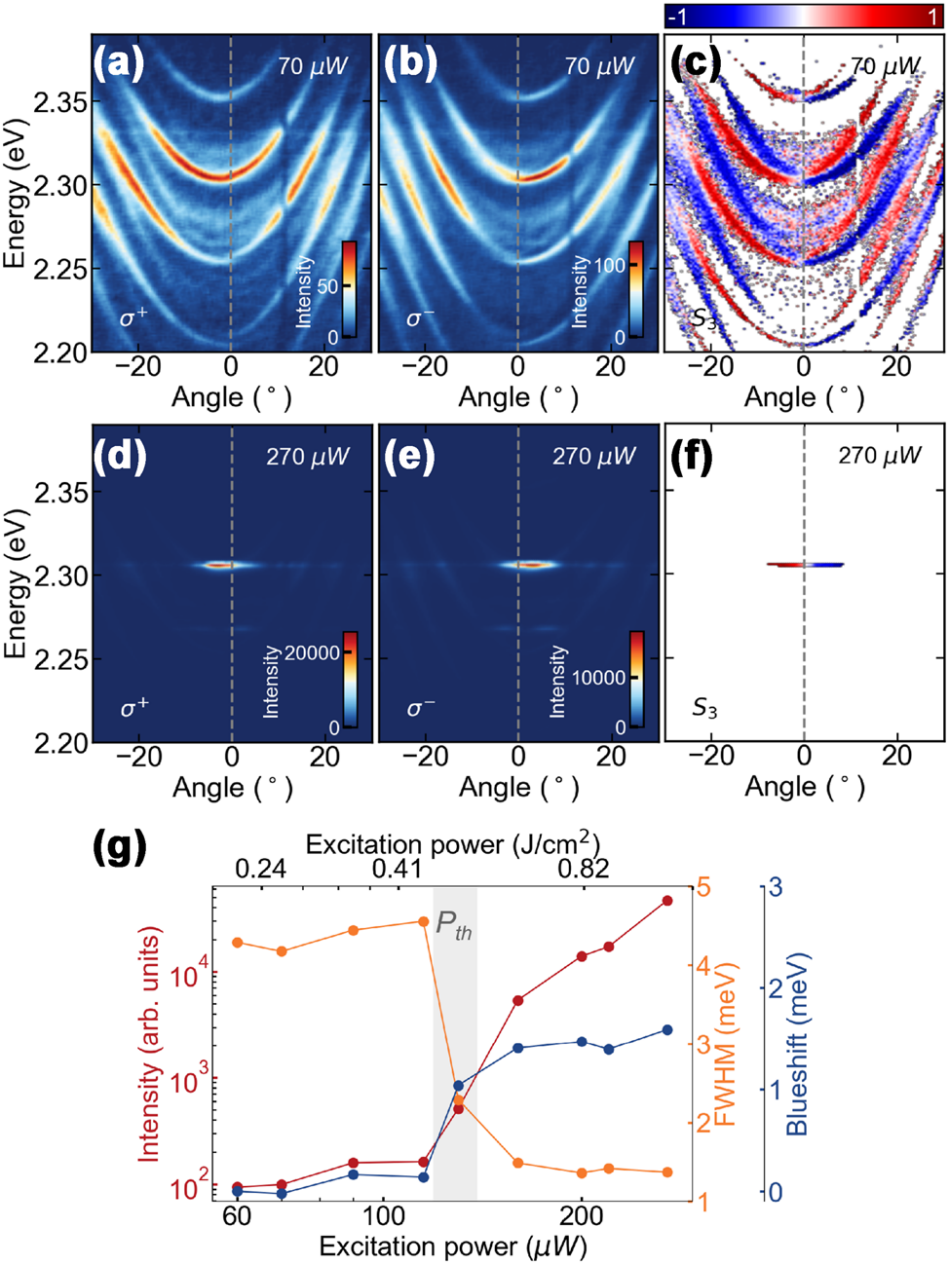
Polariton condensation in the Rashba–Dresselhaus regime. Angle-resolved PL spectra in both circular polarizations: (a and d) *σ* ^+^, (b and e) *σ* ^−^ and (c and f) resulting the *S*_3_ parameter: (a–c) below and (d–f) above the condensation threshold. (g) Emission intensity, spectral linewidth and energy blueshift for increasing excitation power.

Polariton condensation in the Rashba–Dresselhaus SOC regime can be observed in Figure 5. To reach this regime, an external voltage is applied to the device to obtain degeneracy between photonic modes of different parity [47]. Angle- and polarization-resolved PL below the condensation threshold is shown in Figure 5(a and b). The circular polarization of the branches and their separation in emission angle can be observed more clearly by plotting the third Stokes parameter in Figure 5(c) [ $S_{3}=\left(I_{\sigma^{+}}-I_{\sigma^{-}}\right)/\left(I_{\sigma^{+}}+I_{\sigma^{-}}\right)$ , where $I_{\sigma^{+}}$ $\left(I_{\sigma^{-}}\right)$ is the intensity of *σ* ^+^ (*σ* ^−^) circularly polarized light].

With increased excitation power to 130 μW (Figure 5(d–f)) two circularly-polarized condensates form at nonzero and opposite emission angles **k**_‖_ ≠ 0, corresponding to the energy minima of the Rashba–Dresselhaus dispersion relation given by an equation of the form (1). The two momentum-separated condensates exhibit high degree of circular polarization with *S*_3_ equal to 0.75 (0.84) for the *σ* ^+^ (*σ* ^−^) component. The observed dependence of the emission intensity, spectral linewidth and the energy of the condensate on the excitation power, shown in Figure 5(g), follows the typical behavior of a polariton condensate. The observed threshold power equal to 160 μW is approximately two times higher than in the situation without the external electric field.

Polariton condensation is typically regarded as a non-equilibrium process, limited by kinetics. When the mean polariton occupation at some state exceeds unity ⟨*N*_±,**k**_⟩ > 1 due to spontaneous scattering from higher energy states, bosonic stimulation takes over and the condensate starts building up. When the linearly polarized modes of the cavity are well energy-separated, the condensation process occurs into the bottom of the H-polarized mode as we demonstrated in the previous section (see Figures 1(a) and 4). When the cavity modes experience the effective SOC of the Rashba–Dresselhaus type, the dispersion relation exhibits two minima that correspond to the opposite handedness of the emitted light, as shown in Figures 1(b) and 5. Since both spin valleys are degenerate and scattering of polaritons is dominantly spin-conserving, they are equally populated on average with increasing excitation power. To reach the bosonic stimulation regime, occupation of both of those states must approach unity at the same time, which increases the condensation threshold when compared to a situation in which a single mode can drain most of the gain provided by the excitation beam.

## 7 Conclusions

In summary, we presented a versatile and simple solution process method that allows obtaining crystals with controlled dimensions for applications in microavity polaritonics. Unlike other growth techniques, which often require multi-step processes, high temperatures and do not always guarantee size customization, the described approach enables easy control of crystal thickness (ranging from a few hundred nanometres to tens of microns) and width (from a few microns to tens of microns) by simply changing the PDMS template. Furthermore, the technique used in our work has a crucial advantage: the crystals obtained have predetermined shape and position and do not grow randomly on the substrate. This is a very important aspect in view of the integration of these materials into photonic and/or electronic circuits.

The perovskite prepared in this way, was placed in an optical microcavity filled with a nematic liquid crystal. The high optical quality of the material, in conjunction with a strong coupling regime between excitons in the perovskite and photonic cavity modes, allowed for the creation of polariton condensates at room temperature. In our device, the laser-like emission from the condensate can be spectrally tuned by 29 meV, thanks to the sensitivity of the liquid crystal to an external voltage. In addition, we used birefringence of the liquid crystal to induce the Rashba–Dresselhaus spin–orbit coupling. It resulted in locally chiral polaritonic modes and – above the threshold – in simultaneous emission from the polariton condensate in two, tilted beams exhibiting circular polarization of opposite handedness. As the different regimes of spin–orbit interactions differ in the condensation threshold, we showed that it can be additionally operated as an electrically controlled switch of the emission.

## 8 Methods

### 8.1 Synthesis of monocrystals of inorganic perovskite CsPbBr_3_

Single crystals were obtained by slow microfluidics-assisted crystallization from a perovskite precursor solution. A 0.42 M solution was prepared from the mixture of reagents: 106 mg of CsBr and 183.5 mg of PbBr_2_ (Molar ratio 1:1) were added to 1.2 ml of DMSO. This solution was mixed at 80 °C on the hot plate in a glove box, in a nitrogen atmosphere for 2 h. The resulting transparent-clear solution was taken from the glove box, where perovskite single crystals were subsequently crystallized under atmospheric conditions. The crystals were crystallized in microwire-shaped polydimethylsiloxane (PDMS, Sylgard 184, Dow-Corning) templates made by a conventional soft lithography process. In this case, two types of microchannels were used: a single wide rectangular channel 150 μm wide and 2 μm high and a network of microchannels 15 μm wide and 500 nm high. A volume of 3 μl of transparent perovskite precursor solution is deposited at the inlet of the PDMS template, patterned with a single microchannel and a network, and placed in conformal contact with a 8 DBR SiO_2_/TiO_2_ dielectric mirror (SiO_2_ on top) with ITO (indium tin oxide). After the appearance of fine perovskite crystals, the sample is kept in a box, opportunely closed with parafilm, and then transferred to a refrigerator for 24 h and stored at a constant temperature of 2 °C. The presented crystals crystallized after 3 days, during which two consecutive days the process was carried out under atmospheric conditions and room temperature but without the sample’s access to light.

### 8.2 Photoluminescence measurements

The photoluminescence spectra were excited nonresonantly at 2.95 eV (420 nm) (first high-energy reflectance minimum of the structure) with sub-150 fs pulses generated by an amplified Ti-sapphire system working at a repetition rate of 5 kHz. The incident laser beam was focused on the sample using a 50 × microscope objective with NA = 0.65. The emitted light was collected with the same objective. The angular resolution of the emission signal was obtained by imaging of the Fourier plane of the collecting objective onto an entrance slit of a spectrometer equipped with a CCD sensor.

### 8.3 Voltage applied to the cavity

Driving of the LC molecules was performed with a square waveform with frequency of 1 kHz and variable amplitude from a function generator.

## Supplementary Material

Supplementary Material Details
