# Supplementary material for: Electrical polarization switching of perovskite polariton laser
Source: Nanophotonics. 2024 Feb 19;13(14):2659–68. doi: 10.1515/nanoph-2023-0829 (PMC11636435; doi:10.1515/nanoph-2023-0829)
Supplement: Supplementary file 1 — Supplementary Material Details [file j_nanoph-2023-0829_suppl_001.pdf]

# Electrical polarization switching of perovskite polariton laser: Supplementary materials

Karolina Łempicka-Mirek [1] Mateusz Król [1] Luisa De Marco [2,*] Annalisa Coriolano [2,3]
Laura Polimeno [2] Ilenia Viola [4] Mateusz Kędziora [1] Marcin Muszyński [1]
Przemysław Morawiak [5] Rafał Mazur [5] Przemysław Kula [6] Wiktor Piecek [5]
Piotr Fita [1] Daniele Sanvitto [2] Jacek Szczytko [1] and Barbara Piętka [1,†]

[1] *Institute of Experimental Physics, Faculty of Physics, University of Warsaw, ul. Pasteura 5, PL-02-093 Warsaw, Poland*
[2] *CNR NANOTEC, Institute of Nanotechnology, Via Monteroni, 73100 Lecce, Italy*
[3] *Dipartimento di Matematica e Fisica E. De Giorgi, Università del Salento, Campus Ecotekne, Via Monteroni, Lecce 73100, Italy*
[4] *CNR-NANOTEC, Institute of Nanotechnology, UOS Rome, SLIM Lab c/o Dip. Fisica, Università "La Sapienza", Piazzale A. Moro 2, 00185 - Rome, Italy*
[5] *Institute of Applied Physics, Military University of Technology, Warsaw, Poland*
[6] *Institute of Chemistry, Military University of Technology, Warsaw, Poland*

## SEM images

Figures S1 and S2 present images from scanning electron microscope (SEM) of the $CsPbBr_3$ monocrystals grown in channels with thickness of 2 µm and 500 nm. All of the samples were prepared on glass substrates.

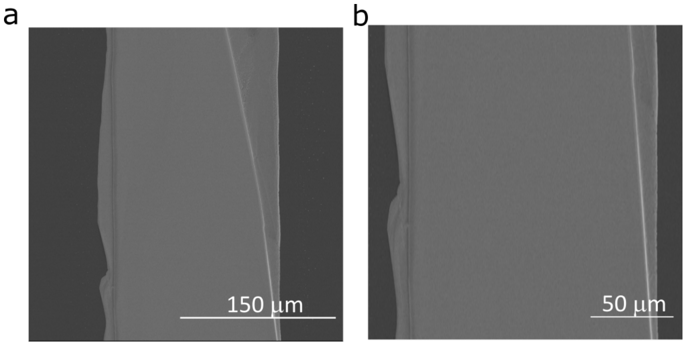


**Fig. S1** SEM images of $CsPbBr_3$ perovskite grown in 2 µm thick channels on glass.

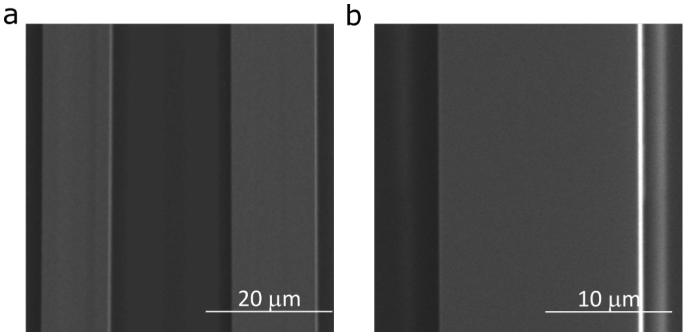


**Fig. S2** SEM images of $CsPbBr_3$ perovskite grown in 500 nm thick channels on glass.

* luisa.demarco@nanotec.cnr.it
† Barbara.Pietka@fuw.edu.pl

## Photoluminescence mapping

To test the homogeneity of the crystal, we performed photoluminescence mapping of a microwire grown in 500 nm thick template in real space. The emission was excited nonresonantly with continuous wave 488 nm (2.540 eV) laser with the approx 10 μm spot size. Fig. S3(a) presents spatial distribution total emission intensity. Fig. S3(b)–(d) shows the emission spectra collected at three different positions on the elongated perovskite crystal, marked in Fig. S3(a) with colored dots.

In addition, Fig. S4 presents comparison between the reflectance spectra and the emission: Fig. S4(a),(b) for 500 nm and Fig. S4(c),(d) for 2 μm thick $CsPbBr_3$ perovskite crystals.

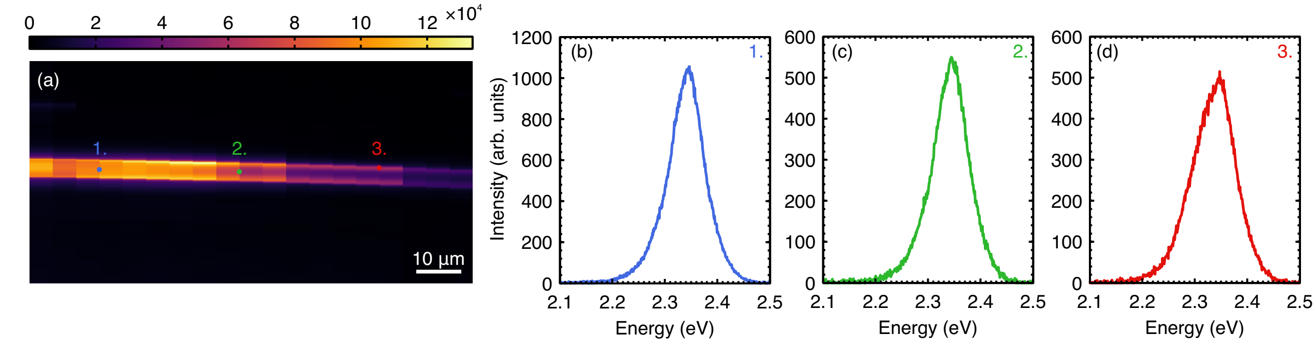


**Fig. S3** Photoluminescence mapping. (a) Total emission intensity. (b)–(d) Emission spectra collected at different positions marked in (a).

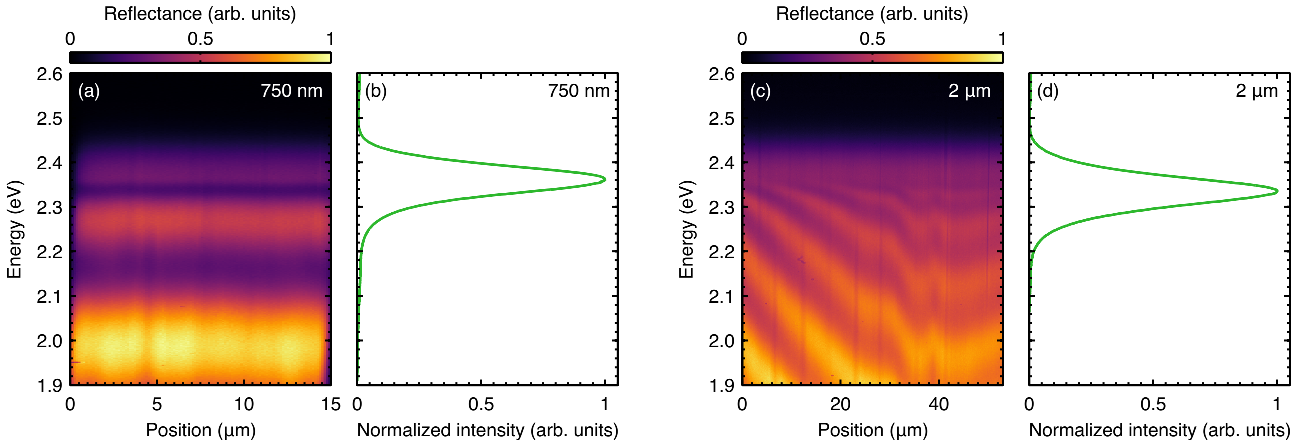


**Fig. S4** Optical characterization of perovskites grown in 500 nm thick templates: (a) normalized reflectance and (b) photoluminescence spectra. Perovskites grown in 2 μm thick templates: (c) normalized reflectance, (d) photoluminescence.

## AFM images

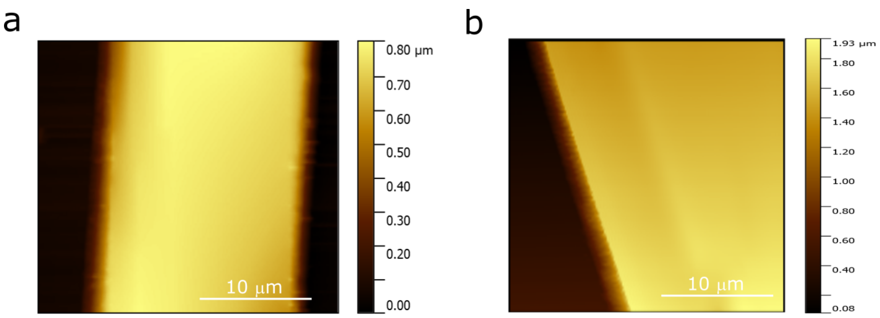


**Fig. S5** AFM topography images of 500 nm-thick (a) and 2 μm-thick (b) $CsPbBr_3$ crystals.

## Confocal characterization

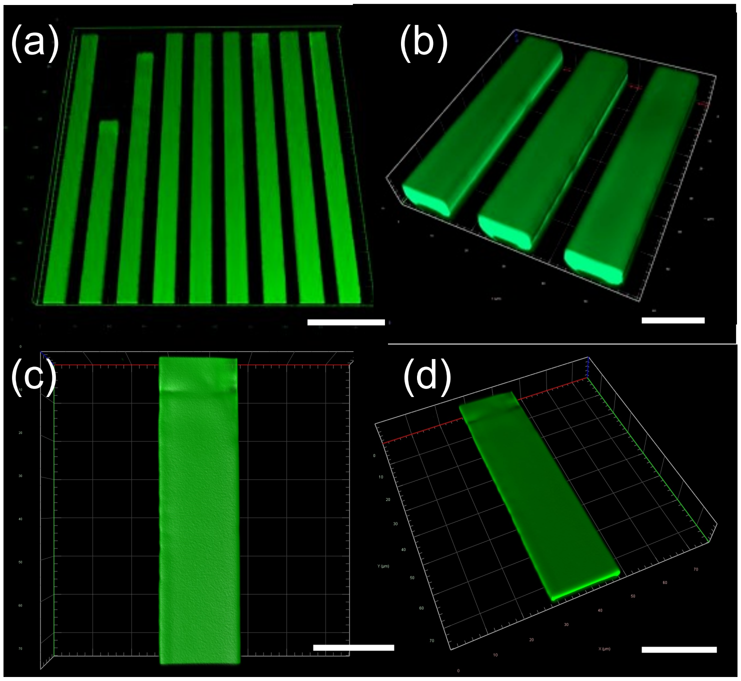


**Fig. S6** 3D z-stack confocal characterizations of a $CsPbBr_3$ mono-crystals realized by microfluidics-assisted crystallization from the perovskite precursor solution. The 3D reconstruction of different crystals are obtained by the confocal acquisition over the crystal's thickness using $\lambda = 405$ nm as excitation laser and 10 μm-wide pinhole (Zeiss-LSM980). (a–b) Network of micro-crystals, 15 μm-wide and 500 nm-high, directly grown on a DBR substrate. Scale bars: 50 μm (a); 20 μm (b). (c–d) Monocrystal, 150 μm-wide and 2 μm-high, on a DBR substrate. Scale bars: 150 μm (c–d). The high-resolution images reveal crystals of good crystalline quality, with controlled and uniform dimensions from the micro to macroscale and homogeneous and smooth interfaces.

## Liquid crystal

The 2091 nematic mixture used in this study is testing custom made medium birefringent material, composed of selected isothiocyanato tolanes and phenyltolanes, having the following properties:
$T_{\mathrm{NI}} = 50.6$ °C;
$\Delta n = 0.3383$ at wavelength of 636 nm, measured using prism coupler technique of the aligned sample;
$n_{\mathrm{o}} = 1.5571$;
$n_{\mathrm{e}} = 1.8954$;

static dielectric data (1 kHz):
$\varepsilon_{\parallel} = 21.24$;
$\varepsilon_{\perp} = 6.03$.
